# Treatment of Non-Hodgkin Lymphoma Involving Head and Neck Sites with a 1.5 T MR-Linac: Preliminary Results from a Prospective Observational Study

**DOI:** 10.3390/hematolrep17020016

**Published:** 2025-03-27

**Authors:** Andrea Emanuele Guerini, Stefania Nici, Stefano Riga, Ludovica Pegurri, Paolo Borghetti, Eneida Mataj, Jacopo Balduzzi, Mirsada Katica, Gianluca Cossali, Giorgio Facheris, Luca Triggiani, Albert Sakiri, Luigi Spiazzi, Stefano Maria Magrini, Michela Buglione

**Affiliations:** 1 Department of Radiation Oncology, University of Brescia, 25123 Brescia, Italy; ludovicapegurri@libero.it (L.P.); paolobor82@yahoo.it (P.B.); e.mataj@unibs.it (E.M.); j.balduzzi@unibs.it (J.B.); m.katica@unibs.it (M.K.); g.cossali@unibs.it (G.C.); giorgio.facheris@gmail.com (G.F.); luca.triggiani@unibs.it (L.T.); albertsakiri@gmail.com (A.S.); stefano.magrini@unibs.it (S.M.M.); michela.buglione@unibs.it (M.B.); 2 ASST Spedali Civili di Brescia, Piazzale Spedali Civili 1, 25123 Brescia, Italy; 3 Medical Physics Department, ASST Spedali Civili Hospital, 25123 Brescia, Italy; stefano.riga@asst-spedalicivili.it (S.R.); luigi.spiazzi@unibs.it (L.S.); 4 Centro per lo Studio della Radioterapia guidata dalle Immagini e dai Biomarkers (BIO-RT), Dipartimento di Specialità Medico-Chirurgiche, Scienze Radiologiche e Sanità Pubblica, University of Brescia, 25121 Brescia, Italy

**Keywords:** MRI-linac, MR-linac, lymphoma, radiotherapy, adaptive, head neck

## Abstract

**Purpose**: Lymphomas are generally radiosensitive; therefore, disease volume tends to shrink during radiotherapy courses. As MRI-linac provides excellent soft tissue definition and allows daily re-contouring of gross tumor volume and clinical target volume, its adoption could be beneficial for the treatment of lymphomas. Nonetheless, at this time there is a lack of literature regarding the use of MR-linac in this context. **Methods**: A prospective observational study was conducted on patients affected by non-Hodgkin lymphoma (NHL) involving head and neck (H&N) sites and treated with Elekta Unity^®^ MR-Linac. The clinical and dosimetric data of the first eight patients were collected and integrated with relevant data from medical records. **Results**: Seven patients had B-cell lymphoma (three DLBCL, two MALT, one follicular, and one mantle-cell) and one T-cell/NK lymphoma. The intent of RT was radical for four patients, salvage treatment for three, and CAR-T bridging for one. Two patients presented orbital localizations and six cervical lymphonodal sites. Median GTV was 5.74 cc, median CTV 127.01 cc, and median PTV 210.37 cc. The prescribed dose was 24–50 Gy in 2 Gy fractions for seven patients and 24 Gy in 3 Gy fractions for one patient. All the patients experienced acute toxicity, the maximum grade was G1 for five patients and G2 for three at the end of RT. One month after radiotherapy seven patients still experienced G1 toxicity, but no toxicity grade ≥ 2 was reported. First radiological assessment was performed for all the patients after a median of 101.5 days, reporting complete response in all the cases. After a median follow up of 330 days, no patient experienced local disease progression, while one patient developed distant progression. **Conclusions**: radiotherapy for NHL with H&N localization using a 1.5 T MR-linac was feasible, with no >G2 toxicity and optimal response rate and disease control.

## 1. Introduction

Although the term lymphoma encompasses an extremely wide and heterogeneous group of hematologic malignancies deriving from lymphocytes, radiotherapy (RT) alone or in combination with systemic therapy is a cornerstone in the treatment of virtually every type of lymphoma [1,2,3].

Progresses in lymphoma treatment allowed an impressive improvement in survival, switching the focus to reducing treatment-related acute and long-term side effects [4,5].

Consequently, modern radiotherapy techniques such as Intensity-Modulated RT (IMRT) and its evolutions (e.g., volumetric and helical treatments, VMAT) and Image-Guided RT (RT) have been extensively investigated and adopted to fulfill this need, allowing the delivery of a more conformal dose distribution, thus sparing the organs at risk (OARs) surrounding target volumes [6,7,8,9].

Currently, radiotherapy workflow is mostly based on computed tomography (CT) imaging for treatment planning and delivery verification. While CT provides valuable anatomical information, it is often limited in its ability to differentiate soft tissues, which is crucial for accurate targeting and dose delivery in complex anatomical regions, including head and neck (H&N) [10].

The recent advent of MRI-guided linear accelerators (MR-Linacs) marked a new era in precision radiotherapy. An MR-Linac is a system integrating a linear accelerator (linac) and an on-board MRI scanner [11]. The benefits of this technology include superior visualization of soft tissue, reducing uncertainties in target and OARs contouring, and the possibility to perform functional studies [12,13].

Moreover, MR-linac allows performing adaptive radiotherapy, as contours of the targets and OARs can be adjusted based on daily MRI, and the treatment plan can be reoptimized and recalculated online for each fraction [14]. As lymphomas are generally radiosensitive and thus prone to volumetric changes during the course of treatment, adaptive radiotherapy could aid in accurately delivering dose to target volumes while minimizing radiation exposure to healthy tissues and potentially reducing treatment-related toxicities [15].

Despite the promising capabilities of MR-Linac technology, there is a noticeable gap in the literature regarding its use for treating lymphoma. No studies have been published to date that specifically address the application of MR-Linac for lymphoma treatment, particularly in the context of its adaptability and precision for managing disease in sensitive regions such as the head and neck. This lack of data highlights the need for clinical research to explore the potential benefits and challenges of integrating MR-Linac into standard lymphoma treatment protocols.

In this paper, we present our initial experience with the Unity MR-Linac system for treating lymphoma at head and neck sites.

## 2. Methods

Clinical and dosimetric data of the first 8 patients affected by non-Hodgkin lymphoma (NHL) involving head and neck (H&N) sites, who were enrolled in a prospective observational study and treated with Elekta Unity^®^ MR-Linac (Elekta Unity, Elekta AB, Stockholm, Sweden), were prospectively collected and integrated with relevant data from medical records.

Immobilization was performed with an MR-safe thermoplastic mask, and a simulation computed tomography (CT) scan was acquired for each patient. Using the same set-up, a simulation MRI was obtained with the 1.5T Ingenia^®^ system (Philips NV, Eindhoven, The Netherlands). Organs at risk (including the oral cavity, eyes, lenses, cochleae, parotid glands, mandible bone, thyroid, and larynx), bones, fat, and air were contoured by a radiation oncologist on a simulation CT scan in order to provide the electron density of tissues. These contours were deformed on a simulation MRI and manually corrected by a radiation oncologist and then used by the medical physicists to generate a synthetic CT. Available diagnostic imaging, including positron emission tomography (PET) CT and/or MRI, was co-registered with the simulation MRI to aid gross tumor volume (GTV) definition. Clinical target volume (CTV) was delineated on the T2 sequence of the simulation MRI according to involved-site RT (ISRT) principles. Planning target volume (PTV) was obtained through an isotropic geometric expansion of 3 mm and cropped 2 mm from the skin. Treatment plans were calculated by medical physicists using 17–19 fields of step-and-shoot IMRT, with the aim to improve plan conformity and homogeneity, and approved by a radiation oncologist. Patients were treated with Elekta Unity^®^ MRI-Linac adopting the adapt-to-shape workflow, in which OARs and target volume contours from the original treatment plan undergo rigid or deformable registration on the daily MRI acquired onboard. Contours can then be manually edited by a radiation oncologist and adapted onto the current anatomy, and the treatment plan is recalculated online by a physicist for each fraction.

## 3. Results

The main characteristics of the patients are summarized in Table 1 and Table 2. Seven patients had B-cell lymphoma (3 diffuse large B-cell lymphoma—DLBCL, two mucosa-associated lymphoid tissue—MALT lymphoma, one follicular lymphoma, and one mantle-cell lymphoma), and one T-cell/NK lymphoma. All the patients presented active sites of disease: two patients had orbital localizations and six cervical lymphnodal sites (in two cases with concomitant nasopharyngeal and in one with concomitant oropharyngeal disease).

The intent of RT was radical for four patients (in the T-cell/NK case followed by chemotherapy), salvage treatment for three (in two cases of refractory disease and in one case for relapse), and CAR-T bridging for one.

Median GTV was 9.9 cc (range 1.14–194.07 cc), median CTV 127.01 cc (range 25.63–496.64 cc), and median PTV 210.38 cc (51.19–709.45 cc). In three cases, two dose levels were prescribed, with volumes for RT boost with a median CTV of 47.77 cc and a median PTV of 90.40 cc.

The prescribed dose was 2 Gy per fraction up to 30 Gy for two patients, 36 Gy with a sequential boost of 4 Gy for another three, 24 Gy for one patient, and 50 Gy for the T-cell/NK case. The patient that received bridging therapy before CAR-T was treated with a dose of 3 Gy per fraction and was the only one that did not complete the planned RT dose, as after 8 of the 10 scheduled fractions, treatment was suspended for G4 febrile neutropenia (not secondary to radiotherapy).

All the patients experienced acute toxicity; the maximum grade during RT course was G1 for five patients and G2 for three. The most common side effects included mucositis (75%, 37.5% G2), dermatitis (50%, all G1), esophagitis (50%, 37.5% G2), xeroftalmia (37.5%, the two patients treated for orbital lymphoma and the patient treated for nasopharyngeal T-cell/NK, all G1), dysgeusia (37.5%, G2 25%), epiphora (G1 25%), and pharyngodynia (25%, all G1). One month after radiotherapy, seven patients still experienced G1 toxicity, but no toxicity grade ≥ 2 was reported. Similarly, three and six months after RT, five patients still experienced grade 1 side effects, with no grade ≥2 toxicity.

The first radiological assessment was performed for all the patients after a median of 101.5 days (in three cases with MRI, in three cases with CT scan, in one with PET-CT and in one with ultrasonography), reporting complete response in all the cases. Therefore, the best overall response was a complete response in all cases.

After a median follow-up of 330 days, no patient experienced local disease progression.

Distant progression was reported only in one case: the patient that underwent RT bridging before CAR-T for r/r DLBCL developed bilateral hilar localizations 13 months after bridging RT.

## 4. Discussion

The use of MR-linacs allows several advantages over conventional linacs, including superior imaging definition for soft tissues and the possibility to perform adaptive radiotherapy. Primary indications could therefore encompass soft tissue sites of disease, remarkably if subject to intra-fraction anatomic variations.

Currently, the most commonly treated sites with this technology include prostate cancer, followed by lymphadenopathies and abdominal (such as hepatic and pancreatic lesions) and pelvic sites of disease [16]. Emerging indications include head and neck cancers and central nervous system cancer.

To the best of our knowledge, up to date only one case report [17] and a case series [18] of two patients have been published regarding the adoption of MR-linac for the treatment of lymphoma. All three case reports regarded treatment of gastric MALT, and adaptive RT using MR-linac allowed margin reductions and resulted in optimal toxicity. This study presents the first cohort of lymphoma patients treated with MR-linac, specifically for head and neck localizations. Our findings indicate an optimal toxicity profile and promising response rates, confirming the feasibility of MR-linac treatment for lymphoma. Our preliminary results compare favorably with those of previous series of H&N lymphomas treated with CT-based RT [19]. All reported toxicities were grade ≤ 2 and were mostly reversible, with only grade 1 side effects observed one month post-radiotherapy. Additionally, no local relapses were identified during a median follow-up period of 330 days. These results suggest that MR-Linac offers a safe and effective treatment option for lymphoma patients, particularly for those with head and neck involvement.

The adoption of MRI-Linac in this setting offers several advantages over “conventional” radiotherapy techniques. Firstly, MRI provides superior image quality for the definition of soft tissues, which is of paramount importance for accurately contouring head and neck disease sites [20].

**Figure 1:**
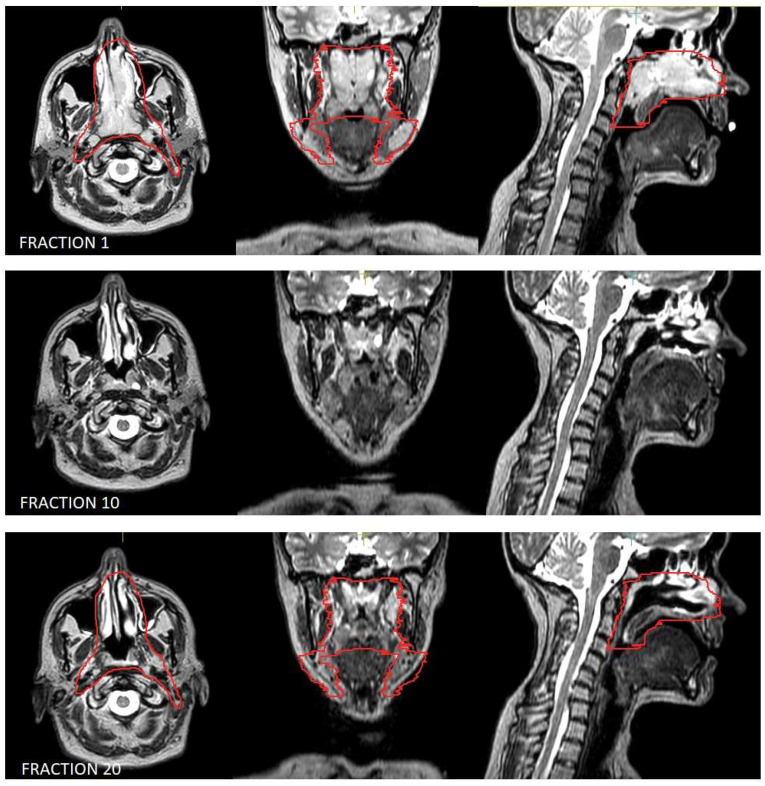
Volumetric variation in disease in the patient treated for natural killer (NK)/T-cell lymphoma, nasal type. Outlined in red is the clinical target volume.

**Figure 2:**
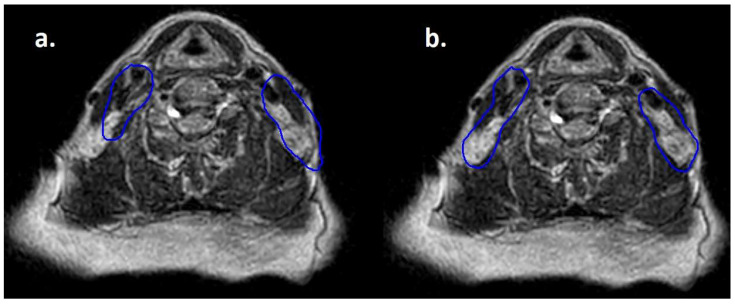
Daily MRI at the sixth fraction of radiotherapy of the patient that received bridging therapy before CAR-T with a dose of 3 Gy per fraction. In (**a**) contours rigidly propagated from simulation MRI; in (**b**) contours deformably registered, manually edited and used for treatment delivery.

Moreover, lymphomas are radiosensitive neoplasms that tend to respond early during the course of radiotherapy, often leading to a substantial volume reduction (as exemplified in Figure 1). While “conventional” CT-based image-guided radiotherapy (IGRT) might allow for the detection of major volumetric variations, MRI-based IGRT enables the identification of even slight differences due to improved soft tissue contrast. Moreover, in our cohort every patient was treated using a daily adapt-to-shape workflow, in which contours of the target volumes and OARs are manually edited for each fraction on the basis of daily MR imaging (as exemplified in Figure 2) and the treatment plan is extensively recalculated. The possibility to adapt contours and the treatment plan on the basis of daily MRI scans could improve dose distribution and guarantee optimal target coverage compared with conventional RT, while sparing surrounding organs at risk [21,22]. This should conceptually result in reduced treatment-related toxicity; nonetheless, clinical data to confirm this hypothesis are awaited (also due to the novelty of this technology).

Another advantage provided by the integration of MRI into radiotherapy image guidance is the possibility to perform functional studies, that could further personalize the treatment based on disease features and response to therapy. Radiomic analyses, for instance, could detect predictive patterns linked with toxicity or disease response [23,24]. This might aid in discriminating early responses from refractory disease, identifying subjects at risk of developing severe side effects, and distinguishing relapse from radiation-induced modifications such as radionecrosis [25]. This could also allow defining a tailored treatment dose; for example confirming, the effectiveness of ultra-low-dose RT for indolent lymphomas, thus reducing the toxicity burden. Immune checkpoint inhibitors have been recently introduced in the treatment of lymphoma, and their indications are steadily increasing due to promising results of clinical trials [26]. In this context, MRI could provide valuable information that could aid in differentiating disease progression from pseudoprogression, which has been frequently identified following immunotherapy [27,28].

However, implementing an MRI-based radiotherapy workflow also poses multiple challenges that must be addressed to optimize therapy. One limitation is the relatively longer delivery time required by MRI-Linac compared to conventional linear accelerators [29].

Moreover, the cranio-caudal length of the radiation field of Unity MR-Linac is limited to 22 cm. Therefore, treatment volumes with larger cranio-caudal extension, which are relatively common for lymphoma, are not eligible for treatment on this system [30].

Similarly, patients with contraindications to MRI (e.g., implantable electronic devices such as pacemakers and implantable cardioverter defibrillators, metallic foreign bodies, cochlear implants, and implantable neurostimulation systems) are not suitable for MR-linac.

Phenomena such as the electron return effect and electron stream effect, which could increase superficial dose and dose at air-tissue interfaces and result in increased toxicities, should also be considered when planning treatment on MRI-linac [31,32]. Furthermore, MR-linac is still an expensive technology due to high installation costs and necessity of specifically trained personnel.

Despite the promising results from this study, its limitations must be acknowledged, primarily the small sample size and the descriptive, observational nature of the analysis. Moreover, in this preliminary cohort, no comparison with patients treated with conventional linacs was planned. The encouraging outcomes observed in this cohort of patients warrant further investigation in larger, prospective studies to validate these findings and better understand the potential role of MR-Linac in the treatment of lymphomas involving head and neck sites.

## 5. Conclusions

Adaptive radiotherapy using a daily adapt-to-shape workflow on a 1.5 MR-linac is feasible for the treatment of head and neck localizations of lymphoma. Optimal results in terms of disease response and toxicity were reported in this case series. Larger prospective studies are awaited to define the role of MR-linac for the treatment of head and neck lymphomas.

## Figures and Tables

**Table 1:** Patients’ characteristics.

Patients’ Characteristics	
**Age**	median 60.3 years (range 48.5–83.2 years)
**Histology**	Diffuse large B-cell lymphoma 3 patients Mucosa-associated lymphoid tissue lymphoma 2 patients Follicular lymphoma 1 patient Mantle cell lymphoma 1 patient T-cell/natural killer cell lymphoma 1 patient
**Stage**	Stage I 6 patients Stage II 1 patient Stage IV 1 patient
**Setting**	First diagnosis 5 patients Relapse 3 patients
**Treatment modality**	Exclusive radiotherapy 4 patients Sequential treatment with chemotherapy 4 patients
**Time from diagnosis to RT**	median 5.45 months after diagnosis (range 1.5–30.7 months)
**ECOG performance status**	PS 0 4 patients PS 1 4 patients

**Table 2:** Characteristics of the disease and treatment before RT.

Lymphoma Type	RT Intent	Stage	Setting	Line of Treatment (*n*)	Systemic Treatment Before RT (*n* of Cycles)
DLBCL MYC-BCL2 “double expressor”	CAR-T bridging	IVA	Relapse	3	R-CHOP (2) + R-COMP (2) + R-miniDHAP (2)
Classic mantle cell lymphoma	Radical RT	IA	First diagnosis	1	no
Natural killer (NK)/T-cell lymphoma, nasal type	radical RT (+ adjuvant CHT)	II	First diagnosis	1	post RT
MALT lymphoma	Radical RT	IA	First diagnosis	1	no
Follicle center lymphoma	Salvage RT	IVA	Relapse	1	no
MALT lymphoma	Radical RT	IA	First diagnosis	1	no
Germinal center DLBCL	PR after I line CIT	IA	Refractory after I line	1	R-CHOP (4) + R (2)
DLBCL MYC-BCL2 “double expressor”	PR after I line CIT	IA	Refractory after I line	1	R-mini CHOP (2) + R (2)

DLBCL = diffuse large B-cell lymphoma; MALT = mucosa-associated lymphoid tissue; CAR-T = chimeric antigen receptor T-cell therapy; RT = radiotherapy; CHT = chemotherapy; CIT = chemoimmunotherapy; R-CHOP = rituximab, cyclophosphamide, doxorubicin, vincristine, and prednisone; R-COMP = rituximab, cyclophosphamide, non-PEGylated liposomal doxorubicin, vincristine, and prednisone; R-miniDHAP = reduced-dose rituximab dexamethasone cytarabine cisplatin; R = rituximab; R-miniCHOP = reduced-dose rituximab, cyclophosphamide, doxorubicin, vincristine, and prednisone.

## Data Availability

The data that support the findings of this study are available from the corresponding author, upon reasonable request.
